# Reprogramming patient-derived tumor cells generates model cell lines for tuberous sclerosis-associated lymphangioleiomyomatosis

**DOI:** 10.18632/oncoscience.375

**Published:** 2017-11-02

**Authors:** Lisa M. Julian, William L. Stanford

**Affiliations:** Ottawa Hospital Research Institute, Regenerative Medicine Program, Ottawa, Ontario, Canada; University of Ottawa, Ottawa, Ontario, Canada; Ottawa Institute of Systems Biology, Ottawa, Ontario, Canada

**Keywords:** lymphangioleiomyomatosis, tuberous sclerosis complex, neural crest, patient cell reprogramming, disease modeling, disease cell of origin

Lymphangioleiomyomatosis (LAM) is a devastating neoplasm characterised by pulmonary infiltration of abnormal smooth muscle-like cells that cause cystic replacement of the lung parenchyma, progressive tissue destruction and ultimately respiratory failure. Renal and uterine tumors as well as infiltration of LAM cell clusters within the lymphatics and venous circulation are also commonly observed, indicative of metastatic potential. LAM is defined by acquisition of inactivating mutations in one of two tumor-suppressor genes: *TSC1* or *TSC2* (herein referred to as *TSC1/2*), best characterized as key negative regulators of mTORC1 signaling[1]. Germline acquisition of a *TSC1/2* mutation leads to development of the multi-system disorder tuberous sclerosis complex (TSC), which includes development of low-grade skin lesions and brain tumors in both sexes and LAM (TSC- LAM) in women. A sporadic form of LAM in women without TSC is caused by somatic *TSC1/2* mutation. Development of LAM lesions is strongly associated with *TSC1/2* loss-of-heterozygosity[1]; mouse models of renal tumorigenesis in LAM have, however, suggested pathogenic roles for *TSC1/2*-heterozygous cells[2,3]. Despite this genetic simplicity, appropriate cellular and animal disease models are lacking for LAM; major barriers have been the inability to propagate patient- derived TSC1/2-deficient LAM cells in culture without immortalization and to generate TSC1/2-deficient animal models that recapitulate pulmonary phenotypes observed in patients. As mouse models of TSC1/2-deficiency within neural stem cell (NSC) populations have supported NSCs as a cell of origin for TSC brain tumors[1], we believe that a better understanding of the lineage that gives rise to pulmonary LAM cells will precipitate the development of effective disease models by identifying appropriate cell culture methods and lineage targeting strategies.

**Figure 1:**
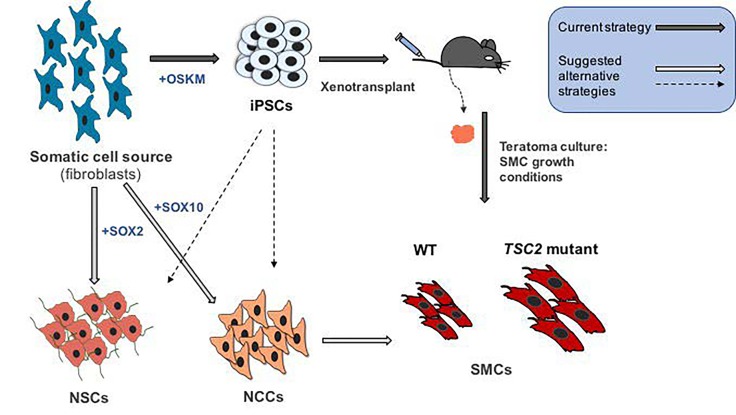
Cellular reprogramming strategies to establish human disease models of LAM and TSC We recently described the establishment of a human LAM cell model[4] in which a somatic cell source (dermal fibroblasts) harvested from either a TSC-LAM patient or unaffected individuals was reprogrammed to iPSCs (via episomal OSKM factor over-expression), and explants of teratoma tumours established from these cells in immune compromised mice were cultured under smooth muscle cell (SMC) growth conditions to establish control (WT) or LAM SMC (*TSC2* mutant) cell lines. *TSC2* mutant SMCs exhibit LAM disease phenotypes such as increased cell size, as depicted. This strategy is amenable to establishing LAM- and TSC-like cells from additional patient-derived somatic cells or from isogenic control and TSC1/2-deficient cells established via strategies such as genomic engineering. Alternatively, TSC and LAM model cell lines could also be established by direct reprogramming of somatic cells to neural stem cells (NSCs) (i.e. by SOX2 over-expression[7]) or neural crest cells (NCCs) (i.e. by SOX10 over-expression[8]), which can be further differentiated to SMCs. NSCs and NCCs can also be differentiated directly from iPSCs.

While the LAM cell of origin is unclear, the fact that LAM lesions are comprised of cells that express markers of the neural crest cell (NCC) lineage, including expression of smooth muscle cell (SMC) markers, suggests a NCC-SMC origin[1]. We recently established a novel cell model of LAM using a patient cell reprogramming approach focusing on the rationale that LAM cells arise from TSC1/2-deficient cells within the SMC lineage[4]. To generate this model, we reprogrammed dermal cultures obtained from either normal-appearing or angiofibroma tumor facial tissue from a TSC-LAM patient (and unaffected human controls) to induced pluripotent stem cells (iPSCs), and subsequently cultured explants of teratoma xenografts generated from these iPSCs under conditions that support SMC growth (Figure 1). The patient-derived dermal cultures possessed a germline *TSC2* mutation (and thus were *TSC2+/−*), with second-hit mutations detected in the angiofibroma cultures at low frequency (and thus were mosaic cultures of *TSC2+/*- and *TSC2−/−* cells). We established expandable cell lines from all patient samples that exhibited multiple features of pulmonary LAM cells compared to controls[4], including expression of NCC smooth muscle and melanocyte markers, elevated mTORC1 activity, deregulated autophagy and metabolic reprogramming. Notably, the angiofibroma-derived iPSCs demonstrated the most potent LAM phenotypes, suggesting epigenetic memory in tumor-derived iPSCs. These are the first non-transformed human cell line models of LAM that can be expanded in culture, and outperform the field's current gold standard human (immortalized) patient-derived cell line in our phenotypic assays. The success of this approach in generating cell lines with extensive LAM-like phenotypes provides strong evidence for the biological identity of LAM cells as aberrant cells of the SMC lineage.

An unexpected but intriguing finding of our study was that at least some *TSC2* expression is required for iPSC reprogramming[4], but not maintenance of pluripotency (unpublished observations). Reflecting this, the iPSCs derived from angiofibroma cultures carried only the germline *TSC2+/−* mutation, which was maintained in the teratoma-derived SMCs. This observation highlighted *TSC2* as a potent regulator of the cell fate decision to adopt a pluripotent state; an important question warranting further investigation is whether *TSC1/2* may also function as critical regulators of fate decisions within the NCC, NCC-SMC and NSC lineages, given that the pathology driven by TSC1/2-deficiency is associated with these cell types[1, 4-6]. The potent LAM phenotypes displayed by our *TSC2*-heterozygous lines offer strong support for the hypothesis that *TSC2+/−* cells contribute to LAM disease progression, even in the absence of *TSC2−/−* cells[2,3]. Our model provides a critical tool to aid in unraveling the biology of LAM cells and to identify therapeutic avenues through drug screening initiatives. Additionally, the generation of TSC2-null iPSCs and human embryonic stem cells via CRISPR-Cas9 mediated genome engineering offers an opportunity to assess the relative contributions of *TSC2*-heterozygous and -homozygous cells to LAM pathology.

Our findings suggest that a TSC-LAM patient cell reprogramming approach can be used more broadly to establish TSC- and LAM-like cell lines from multiple patient biopsies. In addition to our approach of differentiating iPSCs to SMCs in teratomas, iPSCs can be differentiated *in vitro* into putative TSC and LAM cell types of origin (NSCs, NCCs, NCC-SMCs). An intriguing alternative approach that may maintain *TSC1/2*- homozygous mutant cells is direct reprogramming of patient-derived somatic cells into NSCs and NCCs[7,8], forgoing a pluripotent intermediate (Figure 1). Successful reprogramming of TSC1/2-deficient cells and modeling of TSC and LAM phenotypes with these approaches would strongly support the hypotheses that the aberrant cells in TSC and LAM lesions are derived from neural and NC stem cells[1]. Importantly our study, together with recent findings that induction of TSC2-deficiency via genomic engineering in iPSC-derived NSCs can model aspects of TSC brain tumors[5,6], highlights a promising future for the use of stem cell reprogramming, hypothesis-driven differentiation, and genomic engineering approaches to develop more refined cellular models of LAM and TSC, with high likelihood for patient-specific modeling and drug discovery.
